# Calcaneal lengthening using ipsilateral fibula autograft in the treatment of symptomatic pes valgus in adolescents

**DOI:** 10.1186/s12891-021-04855-9

**Published:** 2021-11-23

**Authors:** Chien-Cheng Lai, Ting-Ming Wang, Chih-Hung Chang, Jwo-Luen Pao, Hsu-Wei Fang, Chun-Chien Chang, Shang-Ming Lin, Tsung-yu Lan

**Affiliations:** 1 Department of Orthopedic Surgery, Far Eastern Memorial Hospital, No. 21, Sec. 2, Nanya S. Rd., New Taipei City, 220 Taiwan; 2 Department of Chemical Engineering and Biotechnology, National Taipei University of Technology, Taipei, Taiwan; 3 Department of Orthopedic Surgery, School of Medicine, National Taiwan University Hospital, Taipei, Taiwan; 4 Graduate School of Biotechnology and Bioengineering, Yuan Ze University, Taoyuan, Taiwan; 5 Department of Materials and Textiles, Asia Eastern University of Science and Technology, New Taipei City, Taiwan

**Keywords:** Flexible flatfoot, Evans calcaneal lengthening, Ipsilateral mid-fibula bone autograft

## Abstract

**Background:**

Evans calcaneal lengthening osteotomy is used to treat symptomatic flexible flatfoot when conservative treatment fails. Grafts such as autologous iliac bone grafts, allografts, and xenografts are implanted at the osteotomy site to lengthen the lateral column of the hindfoot. This study aimed to present the outcomes of an autologous mid-fibula bone graft used for calcaneal lengthening in symptomatic pes valgus in adolescents.

**Methods:**

We retrospectively examined 23 ft of 13 adolescents who underwent surgery between July 2014 and January 2018. The radiological and clinical outcomes (American Orthopaedic Foot and Ankle Society ankle-hindfoot scale scores) were assessed during a mean follow-up of 49.7 (range, 30.9–73.4) months. The mean distance of the lengthening site was measured to evaluate graft sinking or collapse. The Goldberg scoring system was used to determine the degree of union at the donor and recipient sites.

**Results:**

The calcaneal pitch and the anteroposterior and lateral talo-first metatarsal (Meary) angles showed significant correction, from 14.4 to 19.6 (*p* < 0.001), and from 14.5 to 4.6 (*p* < 0.001) and 13.5 to 8.5 (*p* < 0.001), respectively. The mean distance of the lengthening site showed no significant change (*p* = 0.203), suggesting no graft sinking or postoperative collapse. The lateral distal tibial angle showed no significant difference (*p* = 0.398), suggesting no postoperative ankle valgus changes. Healing of the recipient and donor sites occurred in 23 and 21 ft, respectively. The American Orthopaedic Foot and Ankle Society ankle-hindfoot scores improved significantly, from 68.0 to 98.5 (*p* < 0.001).

**Conclusions:**

Evans calcaneal lengthening using an ipsilateral mid-fibula bone autograft resulted in significant improvement in clinical and radiological outcomes without ankle valgus deformity. Hence, it could be a treatment option for lateral column calcaneal lengthening in adolescents.

## Background

Pes planovalgus is described as a low or absent medial longitudinal arch with the hindfoot in excess valgus alignment and forefoot abduction [1]. Flexible pes planovalgus refers to the ability of the subtalar joint and longitudinal arch to reverse the alignment. Flexible flatfoot is common in children and usually diminishes with age, and those affected are mostly asymptomatic [2]. Symptomatic flatfoot may present with pain in the sinus tarsi or plantar medial aspect of the midfoot [1, 2]. Most children and adolescents with flexible flatfoot do not require intervention. The indications for surgery include failure of conservative treatment and intractable pain [1, 3]. Operative interventions include osteotomies, arthrodesis, arthroereisis, and soft tissue procedures [1–5].

Evans calcaneal lengthening osteotomy, which was modified by Mosca, has been used to treat symptomatic pes planovalgus [3, 5, 6]. It realigns the foot by lengthening the lateral column and allowing further bone growth. Lateral column lengthening in adolescents with pes planovalgus has good radiological and clinical outcomes, with high patient satisfaction [7]. Graft selection includes autologous iliac bone grafts (AIBG), allografts, and xenografts. The disadvantages of allografts are the lack of osteoinductive and osteogenic properties that may lead to non-union, risk of disease transmission [8, 9], and allograft bone failure [10]. However, in anterior cervical discectomy and fusion, studies have reported the successful use of fibular allografts and instrumentation with acceptable rates of fusion and less postoperative pain [9, 11, 12]. Autologous bone grafts have good osteoinductive and osteogenic characteristics. However, donor site morbidity of AIBG remains a major concern. Studies have shown that non-vascularized autologous fibular grafts used in the reconstruction of bone defects and treatment of non-union of long bones in pediatric patients is a good option [13, 14]. To the best of our knowledge, no study has described the use of autologous mid-fibula bone grafts in Evans calcaneal lengthening.

This study aimed to present the clinical and radiological outcomes, including the degree of autograft incorporation and donor site union, using ipsilateral mid-fibula autograft in adolescents with pes planovalgus.

## Materials and methods

From July 2014 to January 2018, medical records were obtained and retrospectively examined after approval by the institutional review board of our hospital. The inclusion criteria were skeletally immature adolescents and patients with flexible pes planovalgus who were treated conservatively for at least 1 year before surgery. Exclusion criteria were rigid pes planovalgus, cerebral palsy, and endocrine and metabolic diseases. Information regarding the patients’ sex, age at the time of surgery, body mass index (BMI), affected side (right, left, or bilateral), and length of follow-up were obtained by reviewing the medical records.

### Surgical technique

Initially, gastrocnemius release was performed in the musculotendinous junction of the gastrocnemius, as described by Strayer [15], with the patient in the supine position under general anesthesia. The fascial part of the gastrocnemius was released from the medial to the lateral direction. Modified Evans calcaneal lengthening osteotomy, as described by Mosca, was performed by a senior pediatric orthopedic surgeon. A calcaneocuboid (C-C) joint was identified. A 4-cm skin incision was made over the anterolateral region of the distal calcaneus. Dissection was performed, and the sural nerve was protected. Z-lengthening was performed for the peroneus brevis tendon. The C-C joint was temporarily fixed using a K-wire to prevent C-C joint subluxation. The probe was inserted over the dorsal surface of the calcaneus to explore the interval between the anterior and middle facets, and osteotomy was performed through the interfacet interval. The osteotomy was performed 1–1.5 cm proximal to the C-C joint in the anterolateral distal calcaneus. A surgical elevator was inserted in the osteotomy site, and the distal calcaneus fragment (dCF) was raised to correct the abduction of the forefoot. The degree of lengthening under fluoroscopy could be used as a reference for the degree of correction of forefoot abduction.

**Fig. 1:**
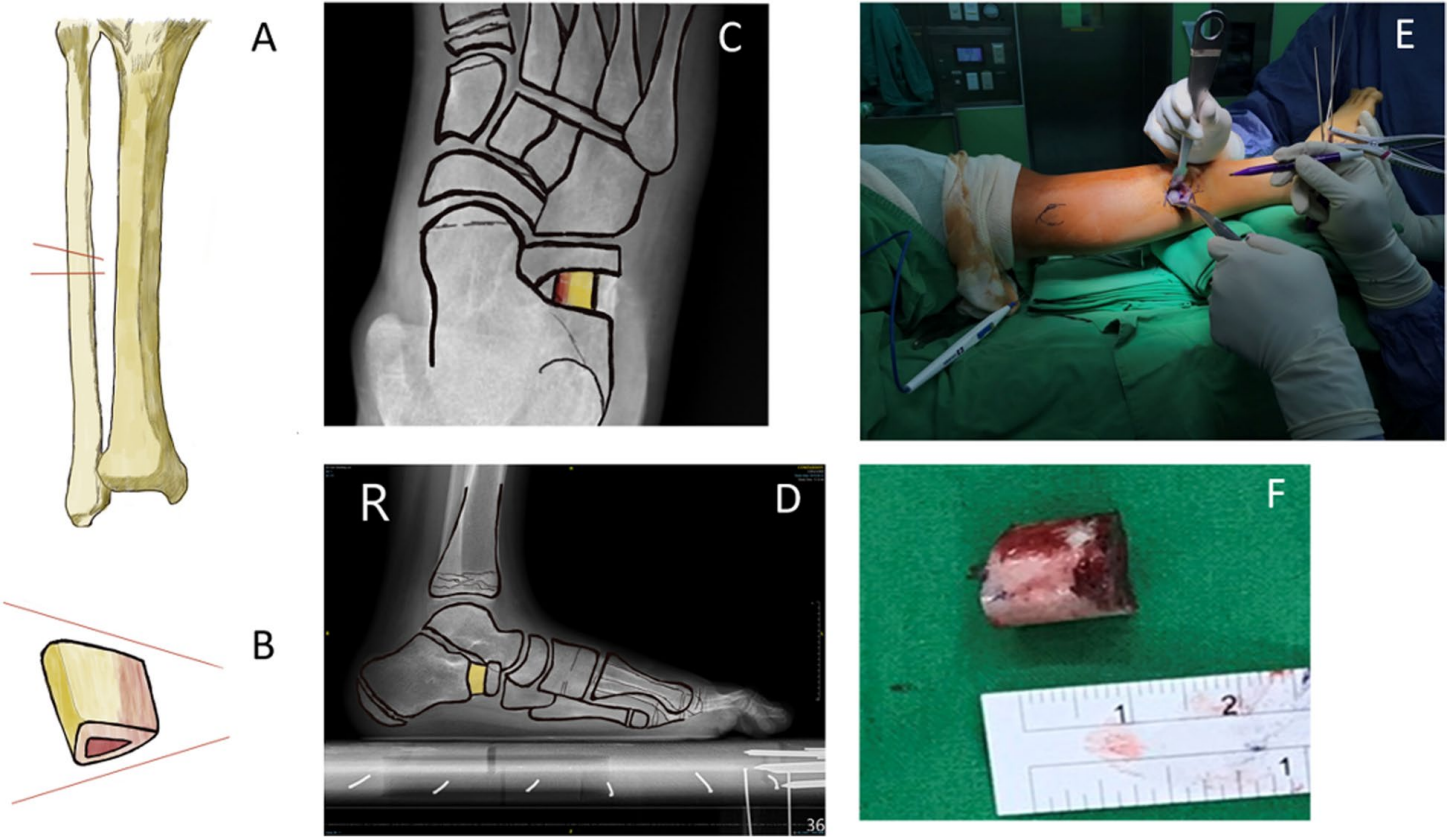
Orientation of autologous fibular graft. **A** and **B** The autologous bone graft harvested from the middle third of the ipsilateral fibula, approximately 1 cm in length and trapezoid in shape. **C** and **D** The orientation of the bone graft in the osteotomy site in the anteroposterior and lateral views, respectively. **E** and **F** The processing of the bone graft harvested and size of graft

A 5-cm skin incision was made over the mid-lateral ipsilateral leg. Dissection was carried out, followed by splitting of the deep fascia. After the deep fascia was split, dissection was performed along the posterior intermuscular septum to avoid injury to the superficial peroneal nerve. This nerve is usually located over the anterior intermuscular septum. The fibula was encountered after the periosteal elevation. The autologous bone graft was harvested from the mid-third ipsilateral fibula and was approximately 1 cm in length and trapezoid in shape (Fig. 1A, B), depending on the degree of correction of forefoot abduction. Two types of saws were used for autograft harvesting: Safedge™ Precision Thin (Stryker, NJ, USA) and an oscillator blade (ConMed, NY, USA). Normal saline cooling was performed when the mid-fibula was sawed. The bone defect of the fibula was filled with a synthetic bone graft substitute, BICERA™ (Hannox International Corp., Taipei City, Taiwan), composed of hydroxyapatite (60%) and β-tricalcium phosphate (40%). The deep fascia of the lateral compartment muscle from the harvest site was closed. The trapezoid-shaped bone graft was impacted to the osteotomy site with the long side of the graft facing the lateral border of the foot. The orientation of the bone graft at the osteotomy site is shown in the AP (Fig. 1C) and lateral views (Fig. 1D). Supplemental 0.062″ K-wires or a pre-contoured locking plate (DARCO™, Wright Medical Technology, TN, USA) fixation was used for stability. The processing of the harvested bone graft and graft size are shown in Fig. 1E and F, respectively. Postoperatively, the foot was protected with a short leg cast with 2 weeks of non-weight bearing and progression to full weight bearing for 4 weeks. After 4 weeks, the cast was removed, and full weight bearing was allowed in normal shoes. Sports activities were allowed after radiographic evidence of bone union.

### Radiographic and clinical function evaluation

Radiographic foot radiographs were obtained using a UT2000 X-ray machine (Philips Research, Eindhoven, Netherlands). All radiographic images were digitally acquired, and radiographic indices were measured using Universal Viewer Zero Footprint® software (GE Healthcare, Barrington, IL, USA). Foot alignment was evaluated by measuring the weight-bearing lateral calcaneal pitch, AP and lateral Meary (talo-first metatarsal) angle, and lateral distal tibial angle (LDTA) (Fig. 1) at 1, 3, 12, and 24 months postoperatively. The LDTA was used to evaluate valgus deformity of the ankle. A small lateral talo-first metatarsal (T-1st MT; Meary) angle indicates less pes planus of the midfoot; a small AP T-1st MT (Meary) angle indicates less abduction of the forefoot and hindfoot. All radiographs were taken using standardized procedures per the guidelines outlined in the textbook of radiology [16]. The normal values of these angles are as follows: weight-bearing lateral calcaneal pitch, 25 °(2 SD range, 15 °to 30 °); AP Meary angle, 10 °(2 SD range, − 10 °to 20 °), and lateral Meary angle, 5 °(2 SD range, − 7 °to 20 °) [17]. The normal LDTA range is 86–92 ° [18]. The fibula donor site and calcaneus lengthening site were also evaluated to examine the degree of union by AP scanography and lateral radiography of the ankle, respectively. All radiographic measurements were performed by three of the authors to assess the interobserver reliability.

The union of the bone graft substitute to the ipsilateral fibula and the autologous bone graft to the calcaneal osteotomy site were evaluated using the Goldberg scoring system, and three categories were chosen [10, 19]. On AP scanography radiographs, the first category evaluates the graft appearance, which gets a score of 0 for resorbed, 1 for mostly resorbed, 2 for largely intact, and 3 for reorganized. The second and third categories evaluated bony union, which received a score of 0 for non-union, 1 for possible union, and 2 for radiographic union at the proximal and distal ends of the graft, respectively. A score lower than 6 at 9 months after surgery was defined as radiographic graft non-union; the highest possible score was 7 points, which represented graft incorporation with excellent reorganization of the graft [10].

The lengthening distance of the calcaneal osteotomy site, where an autologous fibula graft was inserted, was measured using AP and lateral radiographs of the standing foot. Distance α and β represent the longest distance between the distal(dCF) and proximal calcaneal fragments (pCF) under AP and lateral views, respectively (Fig. 1). The mean distance (MD) of lengthening was defined by the following equation: MD = (α + β)/2. Greater MD shortening may indicate graft collapse or graft sinking into the calcaneus [10, 20].

Clinical outcomes were evaluated using the American Orthopaedic Foot and Ankle Society (AOFAS) ankle-hindfoot scale preoperatively and at 12 and 24 months postoperatively. The scoring system consists of nine domains under three different categories—pain, function, and alignment—with a total of 100 points. The visual analogue scale (VAS) was used to assess the ipsilateral fibula donor site at 12 and 24 months postoperatively.

All statistical analyses were performed using SigmaPlot 14.0® (Systat Software Inc., CA, USA). One-way ANOVA and post hoc Bonferroni tests were used to investigate the statistically significant differences between the paired data. Statistical significance was set at *P* < 0.05.

## Results

A total of 25 ft of 15 teenagers were operated during this period. Two feet of two patients were excluded: one because of rigid flat foot and one because of cerebral palsy. A total of 23 ft of 13 teenagers (nine boys and four girls), with a mean age of 12.3 (range, 11–16) years were included in the study. The mean follow-up was 49.7 (range, 30.9–73.4) months. The mean BMI was 23.6 (range, 14.7–35.3) kg/m^2^. Ten patients underwent calcaneal lengthening of the other foot after 7.3 (range, 2.8–11.8) months. K-wire fixation was performed in 4 ft, and locking plate fixation was performed in 19 ft.

### Radiographic measurements

**Fig. 2:**
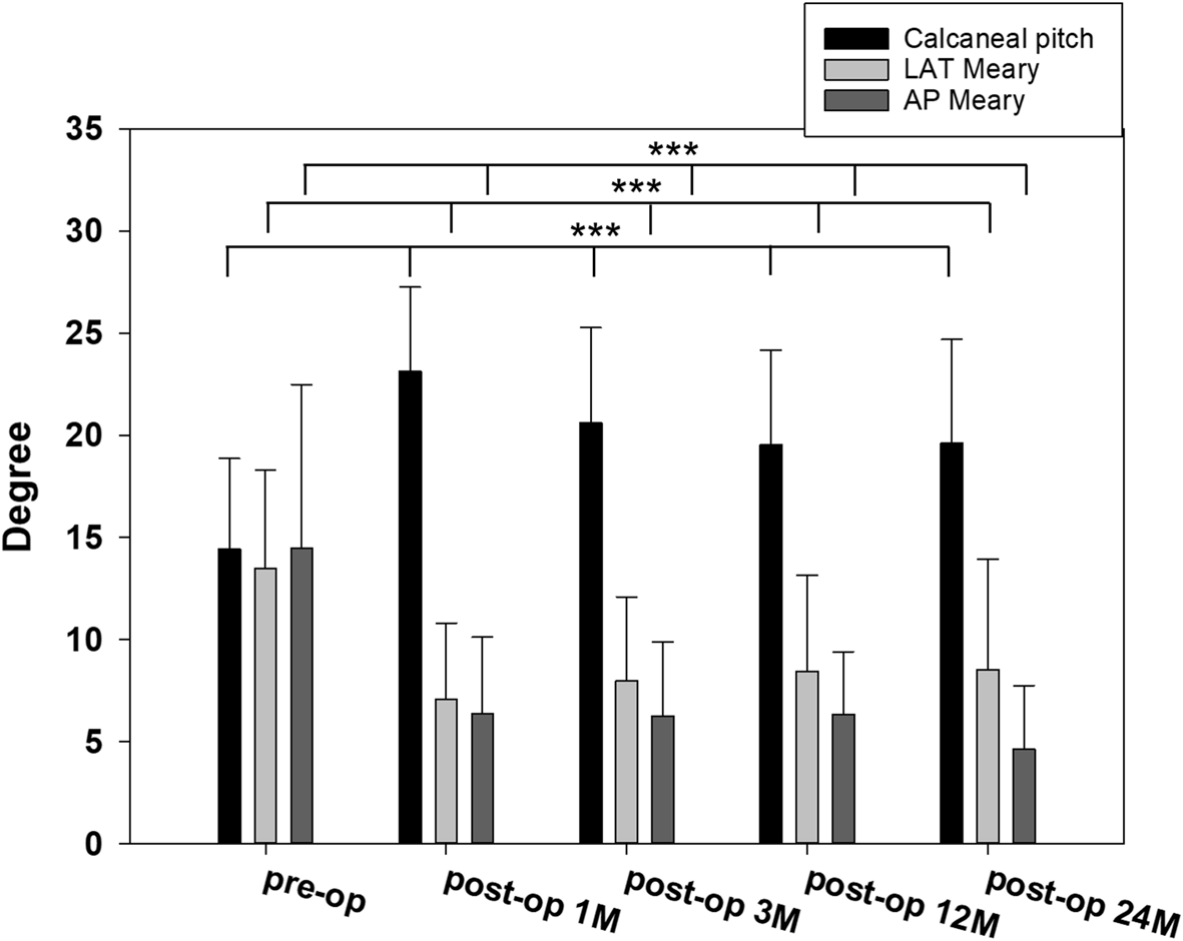
Radiographic measures of preoperative and postoperative. Significant improvement after the operation AP, anteroposterior; LAT, lateral; M, months *p < 0.05, **p < 0.01, ***p < 0.001 vs. pre-op group

Significant improvement was found in the calcaneal pitch, lateral Meary angle, and AP Meary angle at 1, 3, 12, and 24 months postoperatively compared with those preoperatively (Fig. 2). At the 24-month follow-up, a significant increase of 5.2 °in the calcaneal pitch, a significant decrease of 5.2 °in the lateral T-1st MT angle, and a significant decrease of 9.8 °in the AP T-1st MT angle was observed (Table 1). No statistical significance was observed in the LDTA at 12 and 24 months postoperatively, suggesting no ankle valgus deformity in radiographic measurements (Table 1).

**Table 1:** Summary of radiographic measures

	Mean pre-op (Mean ± SD)	Mean 12 M postoperative (Mean ± SD)	*P* value	Mean 24 M postoperative (Mean ± SD)	*P* value
Calcaneal pitch	14.4 ± 4.4	19.5 ± 4.6	< 0.001	19.6 ± 5.1	< 0.001
LAT Meary	13.5 ± 4.8	8.4 ± 4.7	< 0.001	8.5 ± 5.4	< 0.001
AP Meary	14.5 ± 8.0	4.3 ± 3.1	< 0.001	4.6 ± 3.1	< 0.001
LDTA	89.5 ± 1.9	89.1 ± 1.8	0.398	88.7 ± 1.9	0.398

Meary angle indicates talo-first metatarsal angle

*Abbreviations*: *LAT* Lateral, *AP* Anteroposterior, *LDTA* Lateral distal tibial angle, *M* Month

*p < 0.05, **p < 0.01, ***p < 0.001 vs. pre-op group

The MD of the lengthening site was measured, and no significant difference was observed at 3, 12, and 24 months compared with 1 month postoperatively (*p* = 0.203) (Table 2). These findings suggest no graft sinking into the calcaneus or graft collapse at 3, 12 and 24 months postoperatively.

**Table 2:** Mean distance of the lengthening site

	Postop 1 M (Mean ± SD)	Postop 3 M (Mean ± SD)	Postop 12 M (Mean ± SD)	Postop 24 M (Mean ± SD)	*P* value
Mean Distance (cm)	0.9 ± 0.12	0.85 ± 0.12	0.83 ± 0.10	0.86 ± 0.10	0.203

*Abbreviation*: *M* Month

*p < 0.05, **p < 0.01, ***p < 0.001 vs. Postop 1 M group

### Clinical outcomes and complications

The AOFAS ankle-hindfoot scale was used to evaluate clinical outcomes. Significant improvements were observed at 12 and 24 months postoperatively. The scores increased from 68.0 to 97.0 and 98.5 at 12 and 24 months, respectively. The VAS scores for the ipsilateral fibula donor site were both zero at 12 and 24 months.

**Fig. 3:**
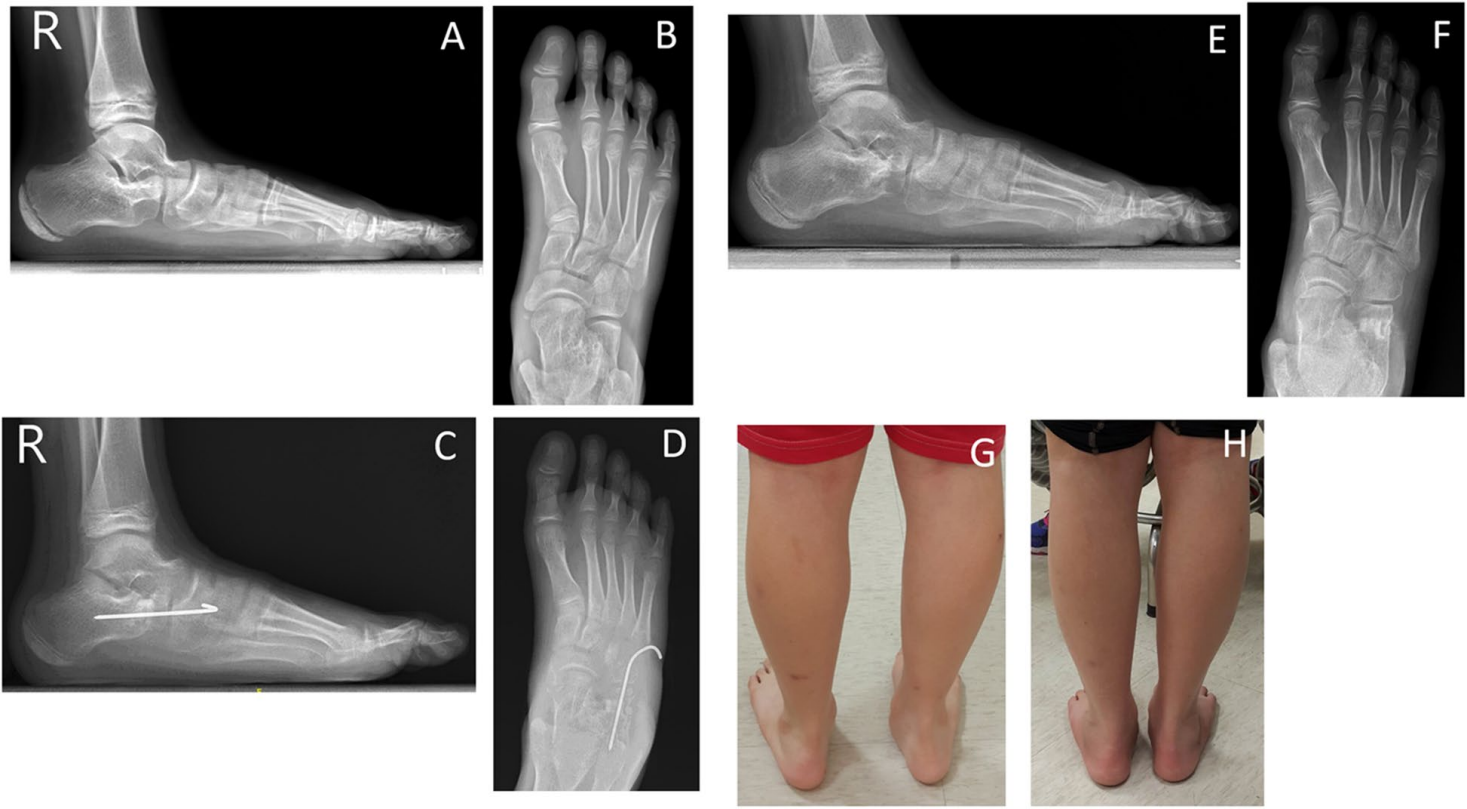
**A** and **B** A 13-year-old boy with symptomatic right flexible flatfoot. **C** and **D** One-month postoperative radiograph showing correction of the calcaneal pitch and the anteroposterior and lateral Meary angles with mild calcaneocuboid (CC) joint subluxation. **E** and **F** Three-month postoperative radiograph showed full union and no graft sinking or graft collapse at the lengthening site. The CC subluxation showed improvement and the clinical outcome was excellent. **G** Preoperative and **H** 3-month postoperative clinical photos

Calcaneal lengthening osteotomy with autologous bone grafting was union in all patients (100%) with mean union time of 2.5 (range, 2.3–3.2) months. The average union time at the ipsilateral fibula autograft donor site was 3.9 (range, 3.2–5.1) months postoperatively, and the union rate was 86.9% at the final follow-up. Figure 3 shows a typical example of this.

Patient 6 (a 13-year-old girl) had asymptomatic nonunion at the bilateral mid-fibula donor site. Patient 2 had a superficial wound infection in one foot 2 weeks after the operation, which resolved after 1 week of oral antibiotics. No other complications, such as calcaneal cuboid joint subluxation, were noted.

## Discussion

The lateral column lengthening technique corrects hindfoot and midfoot deformity and reorientation and localization of the sustentaculum tali to lift the talonavicular joint. A systematic review reported good clinical and radiological results in the medium term, with high patient satisfaction and an acceptable level of complications [7]. However, complications associated with the technique were noted, including nonunion of the osteotomy, dorsal displacement of the anterior calcaneal tuberosity, calcaneocuboid subluxation, overcorrection, undercorrection, relapse of deformity, and injury of the sural nerve and peroneal tendon [7, 21]. Lateral column lengthening has been shown to increase lateral plantar pressure and may be associated with lateral-side foot pain. Lateral plantar foot pain has been reported in 8 to 45% of patients [22, 23]. Pedobarometry studies have shown lateralization of the center of pressure over the hindfoot and midfoot [24]. After lengthening, the increased lateral plantar pressure was measured [25]. In our study, no lateral-side foot pain was observed. Osteoarthritis of the adjacent joints, including the calcaneocuboid and talonavicular joints, was also not found at the final follow-up.

Variants of the original technique, such as Z-osteotomy and double calcaneal osteotomy with minimally invasive surgery, have been reported to minimize complications [26, 27]. However, studies have investigated the incidence and risk factors of allograft bone failure after calcaneal lengthening. The risk of radiographic graft failure, as determined by the Goldberg score, was found to increase with age [28]. Other factors such as sex, affected side of the body (right or left), and ambulatory status were not associated with the Goldberg score. The choice of graft plays an important role in calcaneal lengthening.

Autografts, allografts, and xenografts have been used for calcaneal lengthening osteotomy in the treatment of symptomatic pes planovalgus. Evan used tricortical bone taken from the tibia [29], while AIBG has been used more widely when choosing autologous bone grafts in recent years. A local tricortical autograft obtained from the extra-articular part of the calcaneus was recently reported [30]. Structural bone allografts, including the tricortical iliac crest and patellar and fresh-frozen structural allografts have also been used for calcaneal lengthening [5, 7, 10, 31, 32]. A systematic review reported that structural allografts appear to be at least non-inferior to autologous grafts with respect to the odds of union in hindfoot surgery [33]. However, this systematic review included 10 articles, including adults and children, and most studies have reported hindfoot arthrodesis surgeries. Moreover, there may be some concerns regarding mechanical strength during the processing of allografts, which may result in graft collapse and flatfoot recurrence [34, 35]. The rate of radiographic graft failure using the Goldberg score was 4, and 1% of all patients underwent reoperation [10].

The Goldberg scoring system has been used for frozen bone allograft incorporation [19]. Although computed tomography is a better method for evaluating graft incorporation, it is not covered by the national health insurance in the authors’ country. Therefore, we used the Goldberg scoring system for the incorporation of autologous bone and bone graft substitutes.

Many studies have compared autografts and allografts with different results and complications [32, 36–39]. Autologous cortical bone grafts are incorporated quickly owing to their superior osteoinductivity, osteoconductivity, and biomechanical resistance [39–42]. However, harvesting autologous bone grafts may cause donor-site morbidity. A recent prospective study investigated donor site morbidity after anterior iliac crest bone grafting in 33 children and adolescents (34 hips) [43]. A visual analogue scale score was used to differentiate between the pain at the donor site and that at the recipient site, and a positive correlation was established between the measurements. Pain from autograft harvest was more intense in patients who underwent flatfoot surgery than in those who underwent other corrective osteotomies [20, 43]. In our series, there was no graft collapse, recurrence of flatfoot, and no complaints of donor site pain at the final follow-up.

In weight bearing, the fibula contributes to ankle stability and transmits approximately 17% of the axial load [44]. In previous case-series studies, mid-fibula resection did not cause a biomechanical disturbance in gait and ankle instability [45, 46]. However, donor site morbidity following fibular resection has been reported, including pain, perceived weakness, perceived instability, and paresthesia [46, 47]. In our study, approximately 1 × 1 × 1 cm trapezoidal grafts were harvested from the mid-fibula, and bone graft substitutes composed of hydroxyapatite (60%) and β-tricalcium phosphate (40%) were implanted into the defect. The clinical outcomes were evaluated using the AOFAS ankle-hindfoot scale and were satisfactory at 12 and 24 months of follow-up. The stability domain of the AOFAS ankle-hindfoot scale score was full. Graft subsidence or collapse was not observed in the present study. AP standing scanography also showed no ankle valgus at the final follow-up. However, in our case series, a 13-year-old girl presented with asymptomatic nonunion at the bilateral mid-fibula donor site. Her BMI was 18.7 kg/m^2^, and she had no endocrine or metabolic diseases. She scored 100 points on the AOFAS hindfoot scale at the final follow-up. Pain, perceived weakness, ankle instability, or paresthesia was not observed.

In our study, we used a mid-fibula autograft for calcaneal lengthening. At 1 month postoperatively, the foot radiography of all patients showed some graft incorporation over the lengthening site according to the Goldberg scoring system. Therefore, we allowed full weight bearing at 1 month postoperatively.

To our knowledge, no study has described the use of autologous mid-fibula bone grafts for calcaneal lengthening in symptomatic pes valgus in adolescents. The AIBG and allografts are widely used. However, resorption followed by allograft collapse is also a disadvantage, and revision surgery may be needed later. The reported donor site morbidity and more intense pain when harvesting AIBG are the main concerns. Therefore, ipsilateral fibula cortical autograft could be another choice for calcaneal lengthening surgery.

This study had the following limitations: no control group, small number of patients, shorter follow-up duration, and no kinematic analysis. Our study also lacked clinical measures. A systematic review showed that foot posture index – Six item version (FPI – 6), Staheli arch index or Chippaux-Smirak index, may be the preferred methods of paediatric foot posture measurement [48]. Further investigations and long-term follow-up are required to demonstrate its efficacy.

## Conclusion

Evans calcaneal lengthening using an ipsilateral mid-fibula bone autograft resulted in significant improvement in clinical and radiological outcomes without ankle valgus deformity. Hence, it could be a treatment option for lateral column calcaneal lengthening in adolescents.

## Data Availability

The data used and analyzed during the current study are available from the corresponding author upon reasonable request.

## Abbreviations

AP: Anteroposterior
MD: Mean distance
BMI: Body mass index
AIBG: Autologous iliac bone graft
AOFAS: American Orthopaedic Foot and Ankle Society
T-1st MT: Talo-first metatarsal
LDTA: Lateral distal tibial angle
C-C joint: Calcaneocuboid joint
SD: Standard deviation
dCF: Distal calcaneus fragment
pCF: Proximal calcaneus fragment
